# Extracting Parallel Sentences from Nonparallel Corpora Using Parallel Hierarchical Attention Network

**DOI:** 10.1155/2020/8823906

**Published:** 2020-09-01

**Authors:** Shaolin Zhu, Yong Yang, Chun Xu

**Affiliations:** ^1^Zhengzhou University of Light Industry, Zhengzhou 453000, China; ^2^Xinjiang Normal University, Urmqi 830011, China; ^3^Xinjiang University of Finance and Economics, Urmqi 830011, China

## Abstract

Collecting parallel sentences from nonparallel data is a long-standing natural language processing research problem. In particular, parallel training sentences are very important for the quality of machine translation systems. While many existing methods have shown encouraging results, they cannot learn various alignment weights in parallel sentences. To address this issue, we propose a novel parallel hierarchical attention neural network which encodes monolingual sentences versus bilingual sentences and construct a classifier to extract parallel sentences. In particular, our attention mechanism structure can learn different alignment weights of words in parallel sentences. Experimental results show that our model can obtain state-of-the-art performance on the English-French, English-German, and English-Chinese dataset of BUCC 2017 shared task about parallel sentences' extraction.

## 1. Introduction

Parallel sentences are a very important linguistic resource which comprises much text in the parallel translation of different languages. A large parallel corpus is crucial to train machine translation systems which can produce good quality translations. As is well known, the major bottleneck of statistical machine translation (SMT) and neural machine translation (NMT) is the scarceness of parallel sentences in many language pairs [1–3]. With an increasing amount of comparable corpora on the World Wide Web, a potential solution that alleviates the parallel data sparsity is to extract parallel sentences from comparable corpora. Previous research has shown that this bottleneck can be relieved by extracting parallel sentences from comparable corpora [4–11].

As collecting parallel sentences is important for improving the quality of machine translation systems, many works try to mine parallel sentences from comparable corpora in the last two decades. Their success has a great contribution to the development of this research. Traditional systems developed to extract parallel sentences from comparable corpora typically rely on multiple features or metadata from comparable corpora structure. Bouamor and Sajjad [12] proposed to use a hybrid approach pairing multilingual sentence-level embedding and supervised classifier to identify parallel sentence pairs. They used features such as source-target punctuation marks features and morphosyntactic features to build a support vector machine binary classifier. Although feature engineering is an effective strategy to filter parallel sentences, it usually suffers from the language diversity issue. For example, the named entity is an important feature to measure source-target candidate parallel sentences. However, the named entity has various processes in different languages. For English, CoreNLP (https://stanfordnlp.github.io/CoreNLP/) can be implemented to extract English persons, locations, and organizations, while there are no open-source tools to deal with other lingual named entities such as Uyghur. To address those issues, many methods extracted parallel sentences without feature engineering. More recent approaches used deep learning, such as convolutional neural networks [13] and recurrent neural networks based on long short-term memory (LSTM) [1, 14, 15] to learn an end-to-end network classifier to filter parallel sentences.

**Figure 1:**
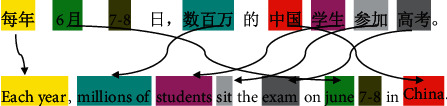
Not all parts of a sentence are equally relevant for representing parallel sentences.

Although mining parallel sentences using neural-network-based approaches has been quite effective, we use the better representations that can be obtained by incorporating knowledge of context information in the model of sentence architecture in this paper. As we all know, not all parts of a sentence are equally relevant for representing parallel sentences (as an example in Figure 1, unmarked words do not affect detecting parallel sentences). That is, different words have various important weights for detecting parallel sentences.

**Figure 2:**
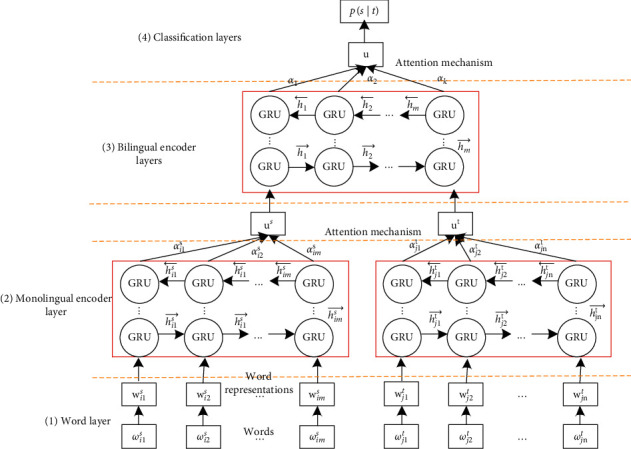
Hierarchical attention neural networks for modeling and selecting parallel sentences.

To address those issues, this paper proposes a parallel hierarchical attention network (PHAN) that learns parallel sentence representations. The PHAN first avoids employing a lot of manual operation to carry out feature engineering. At the same time, compared with current neural networks, the PHAN can effectively learn language differences and the various weights of alignments. As illustrated in Figure 2, the process can be as follows: (1) It first uses one-hot word representations as inputs without feature engineering. (2) Since parallel sentence pairs have different hierarchical components (words form sentences, two monolingual sentences form a parallel sentence pair), the model first encodes monolingual contexts to learn language differences. (3) Then, it inputs those monolingual encodings into a top network to encode a parallel sentence representation. The reason for using this network is that different words in a sentence are different. Moreover, the importance of words is highly context-dependent; that is, the same word may be differentially important in different contexts [2, 16, 17]. (4) Finally, we aggregate the outputs of the neural network into the classification layer to identify parallel sentences. The classification layer adopts the softmax function to implement a binary classification.

Our experimental results show that our method achieves significant and consistent performance compared with all baseline methods in filtering parallel sentences task. In our work, we remove feature engineering and additional computing resources. In particular, we extract parallel sentences from Wikipedia articles. Then, we use the parallel sentences to test the machine translation system and show that the extracting parallel sentences can improve machine translation.

This paper first introduces the main research content.  Section 2 presents a detailed description of the model.  Section 3 presents experiments and settings.  Section 4 gives the detailed results of our experiment. Finally, it is the conclusion of this paper.

## 2. Parallel Hierarchical Attention Network

In this section, we propose a parallel hierarchical attention network (PHAN) to identify parallel sentence pairs. Figure 1 shows the structure of the PHAN. We consider a training parallel dataset *D*={(*S*_*i*_^*s*^, *S*_*i*_^*t*^ : *l*_*i*_), *i*=1,…, *N*} made of *N* pairs of sentences {(*S*_*i*_^*s*^, *S*_*i*_^*t*^)} with labels *l*_*i*_ ∈ {0,1}. If a pair of sentences is parallel, the label is marked as {1}, otherwise as {0}. For example, we set the label of two sentences {^″^I love the motherland^″^, ^″^wo ai zuguo^″^} as {1}.

The network takes a pair of sentences {(*S*_*i*_^*s*^, *S*_*i*_^*t*^)} as input and output is a label of a pair of sentences {*l*_*i*_}. It has two levels, monolingual sentences versus bilingual sentences. The level of monolingual sentences is made of source language encoder and target language encoder. The monolingual encoder is made of two bidirectional GRU (Gated Recurrent Unit) networks with parameters *H*_*w*_ and an attention model with parameters *a*_*w*_, while the bilingual encoder level similarly includes a network and an attention model. The monolingual level mainly encodes monolingual sentence context and dependency. The bilingual level mainly encodes parallel sentence pair interactive context and dependency. The classification layer uses the output *p*(*s|t*) to determine a label {*l*_*i*_}.

### 2.1. Word Layers

In natural language processing, continuous word embeddings [18] are often used as the input of the neural network. However, in this task, we use the one-hot vectors instead of continuous embeddings. The reason for using one-hot vectors is that one-hot vectors can help to encode the context of a sentence. In the first step, to compare source and target sentences in the mathematical sense, we need to project them into one-hot *n*-dimensional space. Each word is converted into a one-hot representation. Although words are often converted into continuous word embeddings, the one-hot representation is more suitable to capture context information.

In order to get this one-hot vector, we define a lexicon *V*={*w*_1_, *w*_2_,…, *w*_*m*_}, where *m* is the number of words of source or target sentences. A one-hot of the word *w*_*i*_ is an array as [0,0,…, 1,…, 0], and we set the number of the word in the lexicon as 1. For example, for a sentence “she is the king,” the lexicon is ['′she'′, '′is'′, '′the'′, '′king'′]. Then, the one-hot of “the” is [0,0,1,0]. The one-hot representation of *j*^th^ word in the *i*^th^ sentence is defined as

(1) $$\begin{array}{c} \omega_{i,j}^{s}=\text{Embedding}\left(w_{i,j}^{s}\right), \end{array}$$

where *w*_*i*,*j*_^*s*^ is *j*^th^ word in the *i*^th^ sentence. *E*^*T*^ is a pretrained embedding matrix, where Embedding( ) is a linear transformational function to embed a word to a one-hot vector. The source language has the same definition.

### 2.2. Encoder Layers

In the above section, we convert words into one-hot word vectors that can be calculated in the neural network. Next, we use a stream-dependent word encoder to encode each word representation to learn the near context information in a sentence.

The traditional recurrent neural network (RNN) is affected by short-term memory. If a sequence is too long, it will be difficult to transfer information into a long step. Therefore, it will miss some important information when we process a long text. For example, when we watch a movie, we may only remember the words such as “amazing” and “excellent” and do not care about the words such as “this,” “is,” and “a” in the next day. The GRU can effectively achieve the above process. It can only keep some relevant information and forget useless data when we obtain parallel sentences. At the monolingual level, in order to learn the information from both directions of words, this paper uses bidirectional GRU to learn the context in a sentence. The GRU used a gating mechanism to track the state of sequences without using separate memory cells. There are two types of gates: the reset gate *r*_*t*_ and the update gate *z*_*t*_. They together control how information is updated to the state. At the time *t*, the GRU computes the new state as follows:

(2) $$\begin{array}{c} h_{t}=\left(1-z_{t}\right)\Theta h_{t-1}+z_{t}\Theta \hat{h_{t}}, \end{array}$$

which is the linear interpolation between the previous state *h*_*t*−1_ and the state $\hat{h_{t}}$ computed with new sequence information. We use the two states to learn the context information in monolingual sentences. The gate *z*_*t*_ decides how much past context information is kept and how much new context information is added. This operation can effectively learn longer context information. *z*_*t*_ is updated as follows:

(3) $$\begin{array}{c} z_{t}=\;\sigma \left(w_{t}x_{t}+\;u_{t}h_{t-1}+b_{z}\right), \end{array}$$

where *x*_*t*_ is the input state sequence vector with time *t*. The other state $\hat{h_{t}}$ is computed in a similar way. $\hat{h_{t}}$ is a corresponding weight that maintains a constant state.

(4) $$\begin{array}{c} \hat{h_{t}}=\tanh\left(w_{h}x_{t}+\;r_{t}\Theta \left(u_{t}h_{t-1}\right)+b_{h}\right). \end{array}$$

In fact, *r*_*t*_ is the reset gate which controls how much the past state information contributes to the sentences. If *r*_*t*_ is zero, then it forgets the previous state. We use the following equation to update the reset gate:

(5) $$\begin{array}{c} r_{t}=\;\sigma \left(w_{r}x_{t}+\;u_{r}h_{t-1}+b_{r}\right). \end{array}$$

In the process, we use *w*_*i*,*j*_^*s*^ to represent a word in a source sentence, *tϵ*[0, *T*]. In order to encode the context information of a sentence, we use the following formula to calculate the hidden representation state for the *t*^th^ time in the source language:

(6) $$\begin{array}{c} \overset{⟶}{h_{i,j}^{s}}=\overset{⟶}{\text{GRU}}\left(w_{i,j\;}^{s}:\theta_{r,t}^{s}\right),\\ \overset{\leftarrow }{h_{i,j}^{s}}=\overset{\leftarrow }{\text{GRU}}\left(w_{i,j\;}^{s}:\theta_{r,t}^{s}\right),\\ h_{i,j}^{s}={\left[\overset{⟶}{h_{i,j}^{s}}|,|\overset{\leftarrow }{h_{i,j}^{s}}\right]}^{T}, \end{array}$$

where $\overset{⟶}{h_{i,j}^{s}}$ and $\overset{\leftarrow }{h_{i,j}^{s}}$ are forward GRU functions and backward GRU functions and *θ*_*r*,*t*_^*s*^ is the model parameter for word GRUs. We obtain the context information for a given word *w*_*i*,*j* _^*s*^ by concatenating the forward hidden state $\overset{⟶}{h_{i,j}^{s}}$ and $\overset{\leftarrow }{h_{i,j}^{s}}$, $h_{i,j}^{s}={\left[\overset{⟶}{h_{i,j}^{s}},\overset{\leftarrow }{h_{i,j}^{s}}\right]}^{T}$, which summarizes information of the whole sentence. Target sentences are encoded like source sentences with an additional neural network layer, which helps the encoder to recognize the most relevant features by emphasizing critical points of the target sentence given by each source sentence.

From the example of Figure 2, we can observe that not all words contribute equally to the representation of the sentence meaning, especially when distinguishing whether two sentences are parallel. Therefore, we introduce an attention mechanism to learn this information that different words have various weights in distinguishing parallel sentences.

(7) $$\begin{array}{c} u_{i,j}^{s}=\tanh\left(h_{i,j}^{s}:\theta_{w}^{s}\right)=\tanh\left(w_{w}h_{i,t}^{s}+\;b_{w}\right)\\ \alpha_{i,t}^{s}=\frac{\exp\left(u_{i,t}^{sT}u_{w}\right)}{\sum_{i=1}^{t}\exp\left(u_{i,t}^{sT}u_{w}\right)},\\ u^{s}=\sum_{1}^{t}\alpha_{i,t}h_{i,t}. \end{array}$$

In the attention process, we first use a one-full-layer perception to learn *u*_*i*,*t*_^*s*^ as a hidden representation of *h*_*i*,*t*_^*s*^. Then, in order to learn the importance of a word in a sentence, we calculate the similarity of *h*_*i*,*t*_^*s*^ with a level context vector *u*_*w*_. Next, we use a softmax function to get a normalized importance weight. Note that *u*_*w*_ is a model parameter in the attention mechanism. The context vector *u*_*w*_ can be seen as a high-level representation that selects which word is more important for a sentence. After that, we get a state *u*^*s*^ by a weighted sum of the word annotations based on the weights. We can get a target vector *u*^*t*^ by the same method.

At the bilingual level, after combining the intermediate vectors *u*^*s*^ and *u*^*t*^, the function networks encode sequence vectors. We concatenate the forward GRU and the backward GRU to obtain the hidden states for each input vector.

### 2.3. Classification Parallel Sentence

In this section, we should detect whether a sentence pair is parallel or not from the top neural network. In order to achieve this goal, we employ a softmax layer to classify parallel sentences. The basic process is that it maps the multiple outputs of the encode layer into an interval (0, 1). In this paper, we treat the classifying parallel sentence as a binary classification problem. We input the source and target sentences into the encode layer. The encoder layer outputs a state vector u into the classification layer. For the classification layer, we use the following formula that maps the input into the interval (0, 1). It is obvious that the output of the classification layer is a probability.

(8) $$\begin{array}{c} l_{i}^{\prime }=P\left(t_{i}|s_{i}\right)=\frac{1}{1+e^{-\left(W_{c}u+b_{c}\right)}}\epsilon \left(0,1\right), \end{array}$$

where *W*_*c*_ is a value matrix and *b*_*c*_ is the bias term for the classification layer. For the classification problem, we usually use the cross-entropy as a loss.

(9) $$\begin{array}{c} l\left(\theta \right)=-\frac{1}{N}\sum_{i=1}^{N}\phi \left(l_{i},l_{i}^{\prime }\right). \end{array}$$

We use *ϕ* to stand for the binary cross-entropy. Then, we use the gold label *l*_*i*_ and predicted label *l*_*i*_′ for a pair of a sentence *i* to optimize the loss. The final objective can be minimized with stochastic gradient descent (SGD) or variants such as Adam to maximize classification.

## 3. Experiments and Setup

In this section, we assess the effectiveness of our model. We compare our method with multiple settings. As we want to improve the performance of our model, we artificially construct negative samples.

### 3.1. Negative Examples

Hangya and Fraser [19] showed that a training model only using parallel sentences is not enough. There are many sentence pairs where the overall meaning is similar, but they are not parallel sentences. So, we need to generate negative examples with similar words but different meanings. Therefore, we generate synthetic noisy data from good parallel sentences. We follow [20] to generate our negative examples that have similar words but different meanings.

Gregoire and Langlais [14] showed that obtaining parallel sentences from nonparallel corpora in practice is an unbalanced classification task in which nonparallel sentences represent the majority class. Although an unbalanced training set is not desired since a classifier trained on such data typically tends to predict the majority class and has a poor precision, the overall impact on the performance of our model is not clear. So, we train a total of 10 models with *kϵ*{0,1,…, 9}, such that with *k*=0 and *k*=9, a model is respectively trained on the dataset with a positive to negative sentence pairs ratio of 100% and 10%.

### 3.2. Data

To implement experiments, we use the BUCC'17 English-French, English-Chinese, and English-German parallel datasets (https://comparable.limsi.fr/bucc2017/cgi-bin/download-data.cgi) to train our model. For test sets, we use the BUCC'17 English-French, English-Chinese, and English-German datasets (https://comparable.limsi.fr/bucc2017/cgi-bin/download-test-data.cgi). Each testing dataset contains two monolingual corpora. The monolingual corpora contain about 100 k–550 k sentences and 2,000–14,000 sentences are parallel. For the convenience of researchers, BUCC 2017 provided us with an evaluation script and a gold standard data to calculate the precision, recall, and *F*-score. For Chinese, we use OpenCC (https://github.com/BYVoid/OpenCC) to normalize characters to be simplified and then perform Chinese word segmentation and POS tagging with THULAC (http://thulac.thunlp.org). The preprocessing of English, French, and German involves tokenization, POS tagging, lemmatization, and lower casing which we carry out with the NLTK (http://www.nltk.org) toolkit. The statistics of the preprocessed corpora are given in Table 1.

### 3.3. Training Settings

We use 256-dimensional GRUs for all RNNs in our model. To prevent the neural network from overfitting, we give the drop-out as 0.5 for the last layer in each module. In order to enhance our model, we add some new negative parallel sentences into training data by sampling {0, 1,…, 9} negative sentence pairs for each parallel sentence pair. For the system, we use TensorFlow to realize our models. All those parameters introduced earlier are based on manual analysis of the data and nonexhaustive tuning on the development set.

### 3.4. Baselines

We compare our model to four baselines (the parameters of the baselines follow their authors):

1. Maximum entropy classifier (ME) [3]
2. Multilingual sentence embeddings (MSE) [12]
3. Dual conditional cross-entropy (DCCE) [21]
4. An LSTM recurrent neural network (LSTM) [14]

The first baseline (ME) is the traditional statistics-based approach that is conventionally considered as alignment features between two sentences. The alignment features mainly conclude the number of connected words, the top three largest fertilities, and the length of the longest connected substring. We use those features to construct a maximum entropy classifier according to Munteanu et al. This method mainly relied on feature engineering. Feature engineering usually suffers from the language diversity issue.

The second baseline (MSE) is an important contribution of this type to approach that mentioned in [22]. First, they used a continuous vector representation of each source-target sentence pair which is learned using a bilingual distributed representation model to reduce the size and noise of the candidate sentence pairs. Then, they filtered source-target sentence pairs by feature engineering and built a support vector machine (SVM) binary classifier to identify parallel sentences. This method also relied on feature engineering.

The third baseline (DCCE): this work proposed dual conditional cross-entropy to extract parallel sentences. This work used the computed cross-entropy scores based on training two inverse translation models on parallel sentences. This method requires additional computational resources to train the translation model.

The final baseline (LSTM) is based on bidirectional recurrent neural networks that can learn sentence representations in a shared vector space by explicitly maximizing the similarity between parallel sentences. This method does not distinguish the various weights of words in detecting parallel sentences. These end-to-end network models do not add attention to encode and do not learn complex mappings and alignments to quantify parallel information.

Compared to the baselines, the PHAN first is independent of feature engineering. It makes the PHAN universal and is easy to apply the PHAN into multiple languages. Moreover, the PHAN uses a parallel hierarchical attention mechanism to capture the deep representation of monolingual and parallel bilingual sentences.

## 4. Results and Discussion

### 4.1. Model Evaluation

In this section, we first give the overall performance of different models.  Table 2 shows precision, recall, and *F*_1_ scores of three language pairs.

From Table 2, we can observe that the two methods of ME and MSE get very poor performance compared with ours. The performance is stable no matter in English-French, English-Chinese, and English-German. As the two methods of ME and MSE rely on feature engineering, alignment and bilingual words need a lot of manual annotation. However, manual annotation only covers limited language information and the high cost of manual annotation makes it difficult to obtain large-scale annotation corpus in many languages or domains. The work of [21] for the WMT18 task performed sentence pairs' extraction, was not feature-based, and gave very good results. We also verify the performance of our method by contrasting [21]. Junczys-Dowmunt [21] trained a multilingual translation model to enforce the agreement of cross-entropy scores. However, they need to train a good machine translation system to improve performance. The trained machine translation system heavily affects the performance of required parallel sentences. From Table 2, we can observe that the results of English-Chinese are not as good as English-French and English-German. As we all know, English-Chinese machine translation is not good as English-French and English-German on the same scale corpus and translation method. The reason is that English-French and English-German are similar languages, but English-Chinese is distant languages. In addition to LSTM, which does not use a parallel attention mechanism, we show a significant increase in our proposed method. Our PHAN outperforms LSTM in three language pairs. We analyze the performance of ours and LSTM; the main difference is that we treat the same words that may be differentially important in different sentences. So, we use two parallel networks and attention mechanism to learn different context information. However, LSTM does not learn this context information as it does not add an effective attention mechanism. Our model uses a parallel attention mechanism to mine more context information to improve performance. In the next section, we will carry out two experiments to further analyze our model.

### 4.2. Qualitative Analysis

We further analyze the performance of PHAN to observe which model can make it perform better than that without the attention mechanism. Alignment is an important factor in identifying parallel sentences. If the weights of alignment are not important, the neural network without attention mechanism may also effectively detect parallel sentences since all alignments have the same contribution. However, the alignment deeply depends on linguistics and context [23–25]. For example, the English word “bearing” means multiple Chinese words such as “chengzhou,” “baochi,” and “zhoucheng” in a different context.

**Figure 3:**
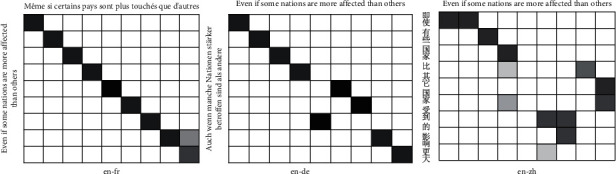
Our results are three alignments in three language pairs.

**Figure 4:**
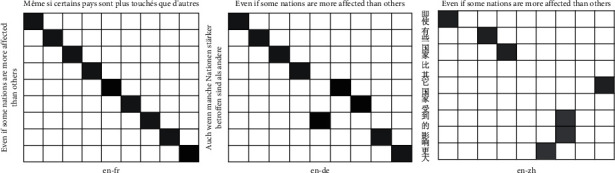
LSTM results are three alignments in three language pairs.

**Figure 5:**
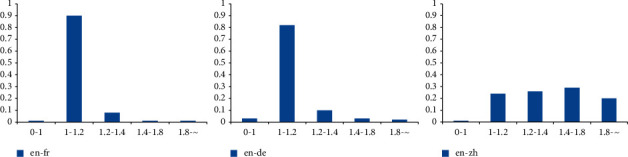
The ratio of the number of words in three language sentence pairs.

We can visualize alignments for some sample sentences and observed translation quality as an indication of an attention model. In order to test that our model is able to mine various informative alignments in parallel sentences, we use this method to make the analysis. To test whether our model can better capture alignments than LSTM without a parallel attention mechanism, we plot the distribution of the attention weights of the words in three language bilingual sentences. The results are shown in Figures 3 and 4. The two figures show that our attention model can obtain a better-visualized alignment. From the two figures, we can find that our model can obtain various alignment weights in three language pairs. For example, our model can distinguish one-to-many alignment in English-Chinese. We can find that LSTM forces the alignment to one-to-one; if a word does not capture alignment, it will not align any words. However, we can observe the alignments of three language pairs; we find that one-to-many occurs more in English-Chinese than English-French and English-German. This may be the main factor that our model gets a bigger improvement in English-Chinese than English-French and English-German. In order to verify this hypothesis, we count the proportion of the number of words in three language sentence pairs. The results are shown in Figure 5. We can observe that English sentences are often longer than Chinese sentences, and the other language pairs have not this situation. This makes one-to-many often occur in English-Chinese. It makes semantic confusion and affects the classification of parallel sentences. This is also an important reason why different language pairs have various accuracies in the classification of parallel sentences.

**Figure 6:**
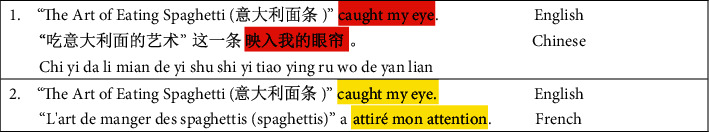
Different languages have different alignments for the same English sentence.

We further explore the language differences and their impact on detecting parallel sentences. We manually extract English-Chinese and English-French parallel sentences to discuss language differences. Example 1 is extracted by the PHAN, but the other baselines miss it. From Figure 6, we can observe that the English phrase “caught my eye” and the Chinese phrase “ying ru wo de yan lian” are not a suitable translation regardless of context information. According to the bilingual lexicon, “Zhua zhu wo de yan jing” is the right translation of the English phrase. However, if we use the translation “Zhua zhu wo de yan jing” to replace the phrase “ying ru wo de yan lian” in the Chinese sentence, the new sentence is wrong. Although the translation is right, it is a wrong collocation in Chinese. The ME, MSE, and DCCE need the lexicon to learn the bilingual signal, which leads to the fact that the word pairs that are not in bilingual lexicon affect detecting parallel sentences. As LSTM has no parallel attention mechanism to effectively encode monolingual information, LSTM cannot encode a monolingual context to distinguish alignments. In fact, language differences and their impact are very important in machine translation. In building machine translation systems, many works add attention to improve machine translation [26]. Example 2 is obtained by all systems. The English phrase “caught my eye” and the French phrase “attiré mon attention” are very right translations in English-French lexicon. From the above, we can conclude that our method can consider language differences by encoding the monolingual context. It can lead to a better result in detecting parallel sentences.

### 4.3. Performance in Machine Translation

In this paper, we hope to obtain parallel sentences and improve the performance of the machine translation system. In the training machine translation system, we use the BUCC'17 English-French, English-Chinese, and English-German parallel datasets as baselines. We use our model to extract parallel sentences from Wikipedia (https://linguatools.org/tools/corpora/wikipedia-comparable-corpora/) corpus. Then, we add the obtained parallel sentences into the three original training data as the new training set for machine translation. To evaluate the translation performance of machine translation, we use the well-known BLEU score. We use phrase-based systems that are trained with Moses for the SMT system. To train the NMT systems, we use OpenNMT (https://github.com/OpenNMT/OpenNMT-py) system.

We trained 48 machine translation systems for each SMT (http://www.statmt.org/moses/) and NMT (https://opennmt.net/) approaches. The baseline systems are trained with BUCC'17 English-French, English-Chinese, and English-German parallel sentences. For the remaining compared systems, we sort the extracted parallel sentence pairs by an extraction system in descending order according to the threshold values and append the top of {20000, 50000,…, 500000} and append the extracted parallel sentence pairs to the original training dataset. We change different numbers of extracted parallel sentences to train the machine translation system to test the stable performance of our model.

Table 3 shows BLEU scores in machine translation systems of SMT and NMT approaches. We can observe that adding the parallel sentences extracted by our model can lead to significant improvement compared to the baseline systems. Therefore, we know that parallel training sentences heavily affect the performance of the machine translation system. This improvement can be observed in three language machine translation systems. The table shows different gains of BLEU scores compared to the baseline systems. When we get Top20K, we add extracted parallel sentence pairs to improve the BLEU score of SMT and NMT systems by 1.13 and 3.1 in English-French, and we also find this improvement in other language pairs. Then, we observe that when we get Top500K, the translation system trained on extracted parallel sentences has better BLEU than Top20K. This means that our model can effectively extract parallel sentences so that it can improve BLEU. We know that adding parallel training sentences can improve the performance of machine translation. These results confirm the quality of extracted sentence pairs and the effectiveness of our model. Hence, we can conclude that our approach could be applied to extract parallel sentences from comparable corpora and improve the performance of machine translation.

## 5. Conclusions

In this paper, we explore a new parallel hierarchical attention network to extract parallel sentences. Our system is able to obtain state-of-the-art performance in filtering parallel sentences while using less feature engineering and preprocessing. Additionally, our model can make full use of monolingual and bilingual sentences. Moreover, we propose a parallel attention mechanism to learn various alignment weights in parallel sentences. In the experiments, we show that our model obtains a state-of-the-art result on the BUCC2017 shared task. In particular, the effectiveness of our model in using the obtained parallel sentences to implement machine translation tasks is demonstrated.

In the future, we will explore the following directions:

1. BPE and similar methods can effectively help us solve the out-of-vocabulary issue. We will use BPE to improve its performance
2. Our model needs parallel sentences to be trained, which can be problematic in low-resource language pairs. In order to lessen the need for parallel sentences, identifying parallel sentences via minimum supervision is a promising avenue, especially in low-resource language pairs

## Figures and Tables

**Table 1:** Training and test set statistics.

Type	Language		Number
Training data	English-French		229,000
	English-Chinese		287,000
	English-German		237,000

Test data	English-French	English	38,069
		French	21,497
	English-Chinese	English	88,860
		Chinese	94,637
	English-German	English	40,354
		German	32,594

**Table 2:** The precision (*P*), recall (*R*), and *F*_1_ scores of extracting parallel sentences.

Model	En-Fr			En-De			En-Zh		
	*P*(%)	*R*(%)	*F*_1_(%)	*P*(%)	*R*(%)	*F*_1_(%)	*P*(%)	*R*(%)	*F*_1_(%)
ME	88.37	83.12	86.72	87.83	82.25	86.02	83.58	80.61	83.90
MSE	93.75	88.43	91.28	92.89	88.05	91.17	90.36	86.93	89.52
DCCE	94.13	89.09	92.45	92.87	89.35	91.78	90.86	87.04	89.82
LSTM	93.89	88.71	92.03	93.05	87.93	91.67	91.83	87.16	90.06
PHAN	94.27	90.03	92.63	93.16	89.73	92.06	92.07	89.37	91.23

**Table 3:** The precision (*P*), recall (*R*), and *F*_1_ scores of extracting parallel sentences.

Data	En-Fr		En-De		En-Zh	
	SMT	NMT	SMT	NMT	SMT	NMT
Baseline	23.71	22.32	21.62	21.35	21.1	17.32
Top20K	24.84 (+1.13)	25.42 (+3.1)	23.38 (+1.76)	25.06 (+3.71)	23.21 (+2.11)	24.56 (+7.24)
Top50K	26.16 (+2.45)	26.35 (+4.8)	24.63 (+3.01)	26.42 (+5.07)	24.66 (+3.56)	25.89 (+8.57)
Top100K	28.31 (+3.6)	27.48 (+5.03)	25.72 (+4.1)	27.67 (+6.32)	25.78 (+4.68)	27.02 (+9.7)
Top200K	29.37 (+4.66)	29.51 (+6.06)	26.76 (+5.14)	28.73 (+7.38)	26.86 (+5.76)	28.13 (+10.81)
Top300K	30.39 (+5.68)	30.55 (+8.10)	27.79 (+6.17)	29.80 (+8.45)	27.91 (+6.81)	29.18 (+11.86)
Top400K	30.41 (+6.70)	30.57 (+9.12)	28.83 (+7.21)	30.82 (+9.47)	28.92 (+7.82)	30.21 (+12.89)
Top500K	31.56 (+7.85)	31.58 (+10.13)	30.14 (+8.52)	31.85 (+10.50)	29.93 (+8.83)	31.22 (+13.9)
